# Supplementary material for: Sweet Basil between the Soul and the Table—Transformation of Traditional Knowledge on Ocimum basilicum L. in Bulgaria
Source: Plants (Basel). 2023 Jul 26;12(15):2771. doi: 10.3390/plants12152771 (PMC10420671; doi:10.3390/plants12152771)
Supplement: Supplementary file 1 [file plants-12-02771-s001.zip › plants-2506249-supplementary.pdf]

Online questionnaire (English translation)

Q1. On what picture do you recognize sweet basil

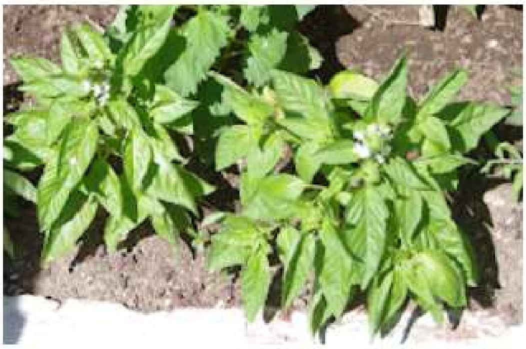

☐ 1

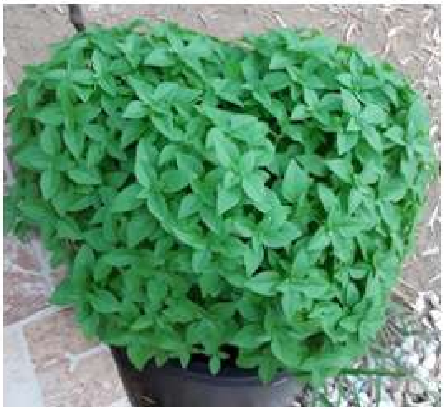

☐ 2

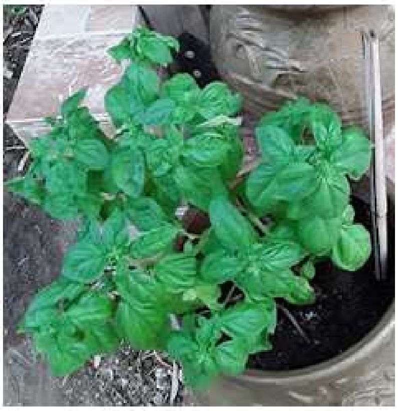

☐ 3

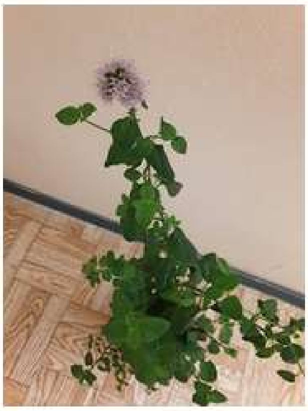

☐ 4

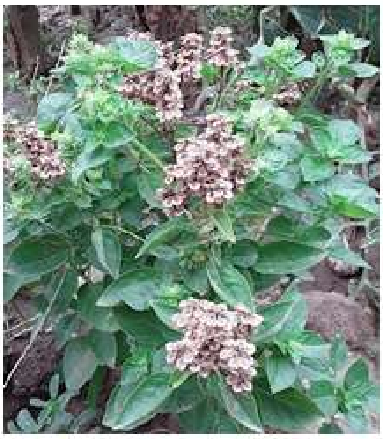

☐ 5

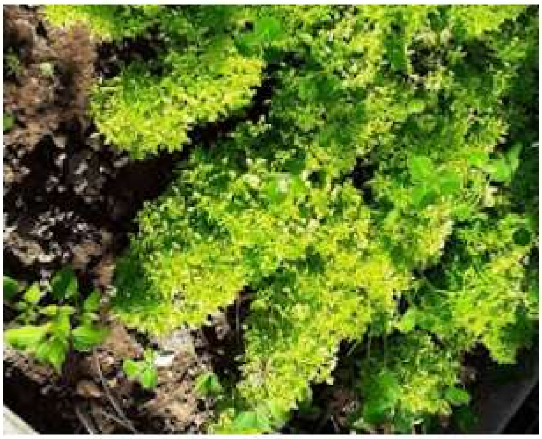

☐ 6

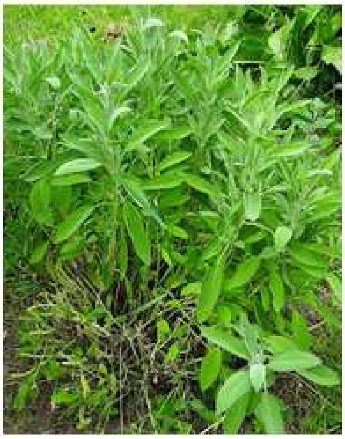

☐ 7

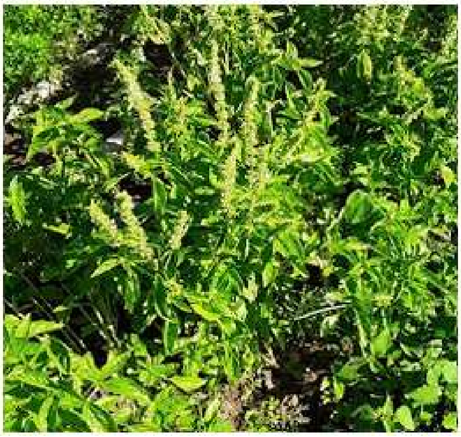

☐ 8

Q2: To me sweet basil is:

- Plant used in different rituals (religious and family ones)
- Medicinal and aromatic plant
- Insecticidal/repellent plant
- Ornamental plant
- Food plant
- Other

Q3. Do you grow sweet basil?

- Yes, in the garden

- Yes, at home in pots or other containers
- No
-

Q4. Why do you grow sweet basil? (Open-end question)

Q5. How do you obtain seeds and/or planting material? (if you grow sweet basil)
- I don't grow sweet basil
- I save my seeds since at least 25 years
- I save my seeds since at least 10 years
- I save seeds but I prefer to change/renew them once every few years
- I buy from the market, preferring Bulgarian producers
- I import/buy imported seeds
- I prefer informal exchange with relatives and friends

Other…….

Q6. Do you distinguish different species/varieties/forms of basil?
- Yes
- I spot differences, but I cannot say what is the difference
- No, I don't distinguish them
- Other……………

Q7. If according to you there are different varieties of basils, are they used in a different manner? (Open-end question)

Q8. Do you think there is Bulgarian/our sweet basil?
- Yes
- No
- I cannot decide

Q9. If you think there is Bulgarian sweet basil, how would you discern it? Write the number of a picture(s) in Q1 that present Bulgarian sweet basil.

Q10. Are you familiar with concept of "Greek" and/or "Italian sweet basil? If, yes, haw do you distinguish them? Are they used for something specific? (Open-end question)

Q11. Do you recognize "Greek" sweet basil on some of the pictures?

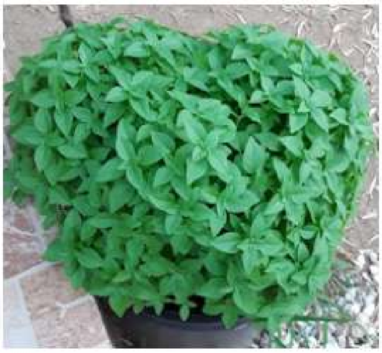

☐ 1

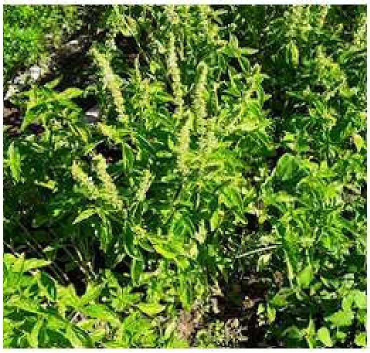

☐ 2

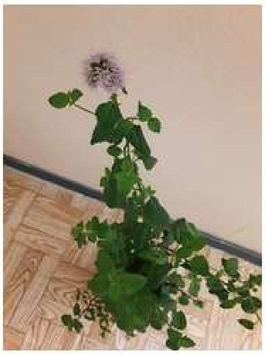

□ 3

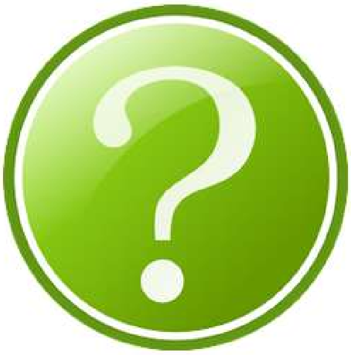

□ None of the above

Q12. Do you recognize “Italian” sweet basil on some of the pictures?

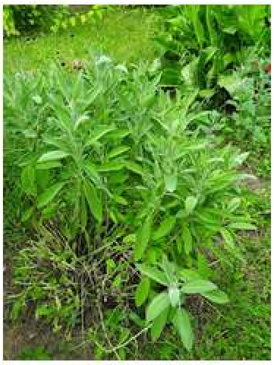

□ 1

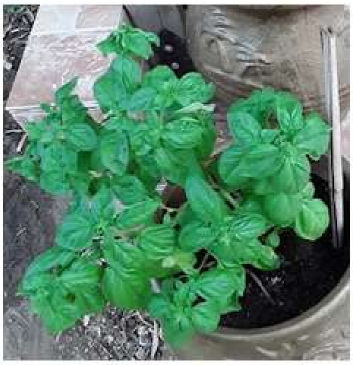

□ 2

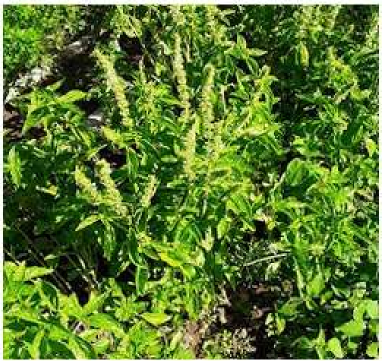

□ 3

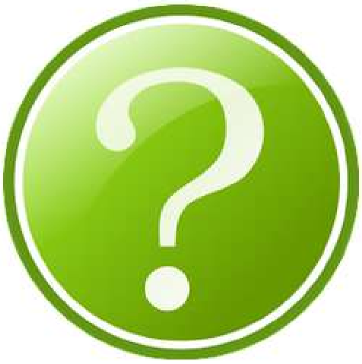

□ None of the above

Q13. Do you have childhood memories about the uses of sweet basil in your home?

- Ornamental purposes – cut flower or in the garden
- Food
- Rituals
- Medicinal/aromatic plant
- Keeping away insects/pest – grown in the garden
- Keeping away insects/pest – dry
- Other………….

Q14. Do you consume sweet basil as food?

- Yes, I know Bulgarian recipes with it
- Yes, I am using it for foreign/international cuisine dishes
- No, I void sweet basil as a food
- Other……….

Q15. Have you used sweet basil in rituals? Have you participated in rituals where sweet basil has central role? (Open-end question)

Sociodemographic parameters

Your age is… (years)

Your sex is… (Female, Male, I prefer to skip this question)

Do you reside in Bulgaria permanently? (Yes/No) – only participants residing permanently in Bulgaria were considered in the calculations.

Your education level is

- Primary
- Secondary
- Secondary (agronomy, agriculture, landscaping, or biological sciences)
- College or university
- College or university (agronomy, agriculture, landscaping, or biological sciences)
- I do not have specialized education but I am keen gardener
- Other……………
